# A Novel Role for Lymphotactin (XCL1) Signaling in the Nervous System: XCL1 Acts via its Receptor XCR1 to Increase Trigeminal Neuronal Excitability

**DOI:** 10.1016/j.neuroscience.2018.03.030

**Published:** 2018-05-21

**Authors:** Emma V. Bird, Tommaso Iannitti, Claire R. Christmas, Ilona Obara, Veselin I. Andreev, Anne E. King, Fiona M. Boissonade

**Affiliations:** a School of Clinical Dentistry, University of Sheffield, Sheffield S10 2TA, UK; b School of Biomedical Sciences, University of Leeds, Leeds LS2 9JT, UK

**Keywords:** aCSF, artificial cerebrospinal fluid, CCI, chronic constriction injury, CGRP, calcitonin gene-related peptide, Cy3, indocarbocyanine, ERK, extracellular signal-regulated kinase, GFAP, glial fibrillary acidic protein, IB4, isolectin B4, Iba1, ionized calcium-binding adapter molecule 1, MAPK, mitogen-activated protein kinase, NDS, normal donkey serum, p38, p38 MAPK, PBS, phosphate-buffered saline, PBST, PBS containing Triton-X, PCR, polymerase chain reaction, pERK, phosphorylated ERK, PFA, paraformaldehyde, pp38, phosphorylated p38, RT–PCR, reverse transcriptase–PCR, TTX, tetrodotoxin, Vc, trigeminal subnucleus caudalis, VGlut1, vesicular glutamate transporter 1, VGlut2, vesicular glutamate transporter 2, vMIP-II, viral CC chemokine macrophage inhibitory protein-II, XCL1, lymphotactin, XCR1, lymphotactin receptor, chemokine, lymphotactin, nerve injury, neuronal excitability, orofacial pain, trigeminal nucleus

## Abstract

Chemokines are known to have a role in the nervous system, influencing a range of processes including the development of chronic pain. To date there are very few studies describing the functions of the chemokine lymphotactin (XCL1) or its receptor (XCR1) in the nervous system. We investigated the role of the XCL1-XCR1 axis in nociceptive processing, using a combination of immunohistochemical, pharmacological and electrophysiological techniques. Expression of XCR1 in the rat mental nerve was elevated 3 days following chronic constriction injury (CCI), compared with 11 days post-CCI and sham controls. XCR1 co-existed with neuronal marker PGP9.5, leukocyte common antigen CD45 and Schwann cell marker S-100. In the trigeminal root and white matter of the brainstem, XCR1-positive cells co-expressed the oligodendrocyte marker Olig2. In trigeminal subnucleus caudalis (Vc), XCR1 immunoreactivity was present in the outer laminae and was colocalized with vesicular glutamate transporter 2 (VGlut2), but not calcitonin gene-related peptide (CGRP) or isolectin B4 (IB4). Incubation of brainstem slices with XCL1 induced activation of c-Fos, ERK and p38 in the superficial layers of Vc, and enhanced levels of intrinsic excitability. These effects were blocked by the XCR1 antagonist viral CC chemokine macrophage inhibitory protein-II (vMIP-II). This study has identified for the first time a role for XCL1-XCR1 in nociceptive processing, demonstrating upregulation of XCR1 at nerve injury sites and identifying XCL1 as a modulator of central excitability and signaling via XCR1 in Vc, a key area for modulation of orofacial pain, thus indicating XCR1 as a potential target for novel analgesics.

## Highlights

• We identified XCR1 in the peripheral and central nervous systems and demonstrated its upregulation following nerve injury.
• In injured nerve, XCR1 is present in nerve fibers, CD45-positive leucocytes and Schwann cells.
• In Vc, XCR1 labeling is consistent with expression in terminals of Aδ- and C-fiber afferents and excitatory interneurons.
• XCL1 increases neuronal excitability and activates intracellular signaling in Vc, a pain-processing region of the CNS.
• These data provide the first evidence that the XCL1-XCR1 axis may play a role in trigeminal pain pathways.

## Introduction

Chemokines are a large family of small, secreted proteins divided into four subgroups – CXC, CC, C and CX3C – according to the number and positioning of the highly conserved cysteine residues in their amino acid sequence (White et al., 2005). They exert their biological effects by binding to cell surface receptors belonging to the G-protein-coupled receptor superfamily, the receptor classes being designated CXCRn, CCRn, XCRn and CX3CRn (Kufareva et al., 2015). Chemokines have a well-established role regulating the migration of leukocytes and coordinating inflammatory responses. An increasing number of studies have demonstrated an important role for chemokine signaling in the nervous system (Rostène et al., 2007), where they have diverse effects in a range of physiological and pathological processes, regulating neuronal development, neuroinflammation and synaptic transmission. Consequently, they have been implicated in a variety of neurological disorders including multiple sclerosis, Parkinson’s, Huntingdon’s and Alzheimer’s diseases (Mines et al., 2007, Hamann et al., 2008, Wild et al., 2011, Cheng and Chen, 2014, Savarin-Vuaillat and Ransohoff, 2007). Some chemokines have also been shown to be critical in the development and maintenance of chronic pain, via their roles in altered excitability and nociceptive processing (Grace et al., 2014, Mélik Parsadaniantz et al., 2015, Tsuda, 2017). Chronic pain, including that from the orofacial region, represents a major health issue, impacting on health and quality of life, and on economic and employment issues (Phillips and Harper, 2011, Patel et al., 2012, Dueñas et al., 2016, Rice et al., 2016). Despite improved knowledge of mechanisms underlying pain, there are still considerable gaps in our understanding of chronic pain – including neuropathic pain – occurring as a consequence of pathology or injury affecting the nervous system. Treatment for this type of pain is limited and often ineffective (Rice et al., 2016). As outlined above, some chemokines act as neuromodulators within the nervous system, where they are expressed by both glia and neurons (Miller et al., 2009, Old and Malcangio, 2012), and thus may provide novel potential therapeutic targets for chronic pain.

Lymphotactin (XCL1) – a member of the least examined C class of chemokines (Kelner et al., 1994) – is produced by subsets of T cells and natural killer cells in response to infection and inflammation, and is chemotactic for T lymphocytes through binding to its receptor (XCR1) (Huang et al., 2001, Dorner et al., 2002, Lei and Takahama, 2012). Initial studies indicated expression of XCR1 within a range of immune cells, however more recent work indicates that it is selectively expressed in subsets of dendritic cells (reviewed in Lei and Takahama, 2012). Increased levels of XCL1 and XCR1 have been reported in joint fluid of rheumatoid arthritis patients (Wang et al., 2004), and XCL1 is also present in oral mucosal endothelial cells in oral cancer (Khurram et al., 2010, Kiaii et al., 2013), suggesting that XCL1 may be present in other cell types and is upregulated in disease conditions. Other reports demonstrate involvement of XCL1-XCR1 in Crohn’s disease (Middel et al., 2001) and in suppression of HIV-1 infection (Guzzo et al., 2013). There is little evidence to date for the presence of XCL1-XCR1 in the nervous system or of its potential role in nociceptive processing. Therefore the overall aims of this study were to investigate the role of lymphotactin and its receptor in hyperexcitability and signaling, and establish their potential contribution to the development of orofacial neuropathic pain.

In this study we used immunohistochemistry to examine which components of the orofacial pain pathway express XCR1. Specifically, we determined the expression of XCR1 in specific cell types at the site of a trigeminal nerve injury and in the trigeminal subnucleus caudalis (Vc), a region of the brainstem involved in the central processing of orofacial pain. Using a combination of pharmacological and electrophysiological studies, we investigated the novel role for XCL1-XCR1 axis in the modulation of increased neuronal activity and altered intracellular signaling (specifically c-Fos, pERK, pp38) within Vc, and determined the potential contribution of this axis to the development of trigeminal neuropathic pain.

## Experimental procedures

### Animals

A total of 47 rats were used in the study: 22 male Sprague–Dawley rats (age 7–9 weeks; 225–250 g); 6 female Wistar rats (age 3–5 weeks; 70–80 g); 3 male Wistar rats (age 7–9 weeks; 200–250 g); and 16 male Wistar rats (age 3–5 weeks; 70–80 g). Details of animal use for different elements of the study are described in the relevant sections below; in all cases animal numbers were based on power calculations using data from previous studies that employed similar protocols. All animals were obtained from Harlan Laboratories Ltd (Bicester, UK). They were allowed to acclimatize to the colony room (Biological Services, University of Sheffield or Central Biological Services, University of Leeds) for at least 7 days after arrival and were housed in polyethylene cages (4 per cage), controlled for temperature (21 °C) and humidity (55%) under a regular 12-h light/dark cycle (lights on 08:00; lights off 20:00). Standard laboratory rodent chow and water were available *ad libitum*. All efforts were made to minimize animal suffering and to reduce the number of animals used in the study. Experimental protocols were performed under appropriate UK Home Office Licences, with local ethical approval, and in accordance with current UK legislation as defined in the Animals (Scientific Procedures) Act 1986. The ARRIVE guidelines (Kilkenny et al., 2010) have been followed in reporting this study.

### Characterization of XCR1 expression in the trigeminal system

#### Mental nerve injury

Adult male Sprague–Dawley rats (*n* = 16; 225–250 g) received a chronic constriction injury (*n* = 8, CCI) to the mental nerve or a sham procedure (*n* = 8, Sham). The CCI procedure has been described previously in studies carried out in our laboratories (Bird et al., 2002, Evans et al., 2014), and the techniques used were in keeping with Bennett and Xie’s original description of the CCI (Bennett and Xie, 1988). Under general anesthesia (isoflurane; 4% induction and 2–3% maintenance) the left mental nerve was exposed and constricted with two loosely tied 6/0 chromic catgut sutures (Ethicon, Norderstedt, Germany), with a spacing of 1 mm between the sutures. In the Sham group the mental nerve was exposed, but no constriction performed. In all animals the subcutaneous tissue and overlying skin were closed with 4/0 vicryl sutures (Ethicon). The animals were left to recover for periods of 3 or 11 days (*n* = 8 [4 CCI, 4 Sham] per recovery period). Naïve adult male Sprague–Dawley rats were also used as unoperated controls (*n* = 4; 225–250 g).

#### Tissue collection

At the end of the recovery period rats were deeply anesthetized with pentobarbital (500 mg/kg, i.p.; J M Loveridge Ltd, Andover, UK) and perfused transcardially with 500 ml phosphate-buffered saline (PBS), followed by 500 ml 4% paraformaldehyde (PFA) fixative. Right and left mental nerves and trigeminal ganglia, and brainstems were removed, post-fixed in 4% PFA for 4 h and cryoprotected in 30% sucrose overnight, both at 4 °C. A small groove was made along the ventral surface of the brainstem to allow identification of the left and right sides. All the tissues were then embedded (brainstem transversely, ganglia and nerves longitudinally) in Tissue-Tek OCT compound (Sakura Finetek, Alphen aan den Rijn, Netherlands) and stored at −80 °C. Frozen serial brainstem sections (30 µm) were sectioned on a microtome cryostat (Microm HM560; Thermo Scientific, Walldorf, Germany), from 5 mm caudal to 10 mm rostral to obex (the point at which the central canal opens up into the fourth ventricle) and collected free-floating in 24-well plates. Mental nerves and trigeminal ganglia were serially sectioned at 14 µm and thaw-mounted onto poly d-lysine (Sigma–Aldrich Company Ltd, Gillingham, Dorset, UK)-coated glass microscope slides.

#### Immunohistochemistry

**Fig. 1:**
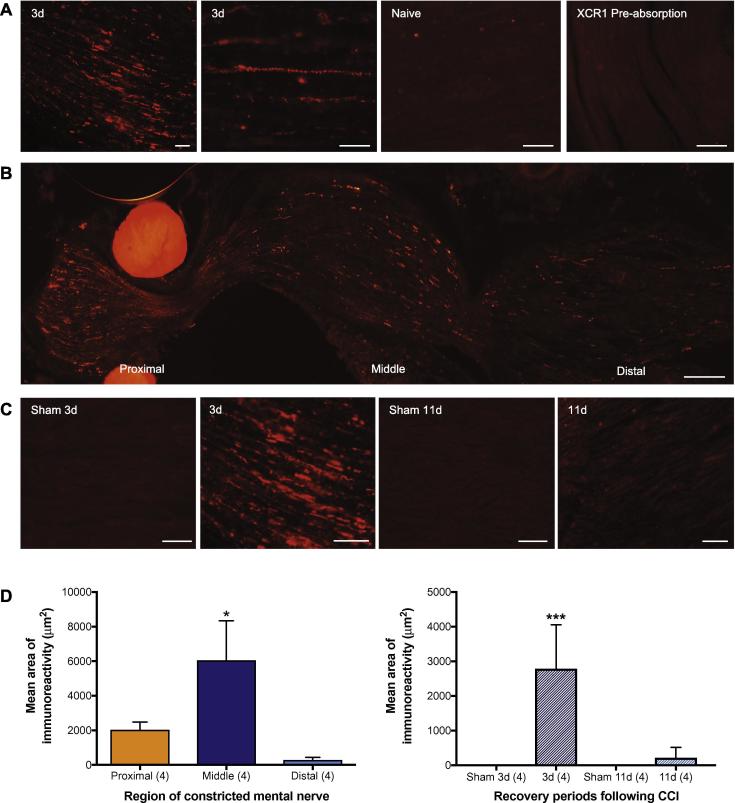
XCR1 is not expressed in naïve or sham-operated rat mental nerve, however it is present following peripheral nerve injury. (A) XCR1 immunofluorescent labeling in rat mental nerve, 3 days (3 d) following chronic constriction injury (CCI) to the nerve (far left and center left) and in an uninjured nerve (naïve, center right), and following preabsorption of the XCR1 primary antibody with its respective hapten. Scale bars = 100 μm. (B) Montage of photomicrographs showing XCR1 expression in the three distinct regions of the mental nerve, 3 days after CCI. The bright red circular structures at the proximal end are the sutures used to perform the CCI. Scale bar = 100 μm. (C) XCR1 immunofluorescent expression appeared to be more abundant 3 days following CCI (3 d) compared with 11 days after injury (11 d) and in sham control mental nerve. Scale bars = 100 μm. (D) Levels of XCR1 fluorescent immunoreactivity were significantly higher in the middle region of the constricted nerve 3 days after CCI compared with the proximal and distal regions (left panel, *p* < 0.05). XCR1 immunoreactivity was higher in 3 days post-injury nerves, compared with 11 days post-injury nerves (right panel, *p* < 0.0001); after 3 days, XCR1 labeling was also significantly higher in the CCI group than in the Sham group (no labeling) (*p* < 0.0001). Data are expressed as the mean ± SEM; statistical analysis by ANOVA with post-hoc Tukey’s multiple comparisons test. Note that the second panel of (C) is a higher magnification image of the same section shown in the first panel of (A).

Free-floating brainstem sections, and nerve and ganglia slides were blocked in PBS containing 0.5% Triton X-100 (PBST) and 20% normal donkey serum (NDS; Jackson ImmunoResearch Labs Cat# 017-000-001; RRID:AB_2337254; Jackson ImmunoResearch Laboratories Inc., West Grove, PA, USA) for 1 h at room temperature and then incubated overnight at 4 °C with rabbit anti-XCR1 primary antibody (1:500; LifeSpan Cat# LS-A158-50; RRID:AB_1116636; LifeSpan Biosciences, Inc., Seattle, WA, USA) diluted in PBST and 5% NDS. They were then incubated for 90 min at room temperature with donkey anti-rabbit secondary antibody conjugated to indocarbocyanine (Cy3) (1:500; Jackson ImmunoResearch Labs Cat# 711-165-152; RRID:AB_2307443; Jackson ImmunoResearch Laboratories Inc., West Grove, PA, USA) diluted in PBST containing 1.5% NDS. If no further labeling was required (i.e. XCR1 alone), tissue was mounted and coverslipped using fluorescence-free Vectashield medium (Vector Laboratories, Burlingame, CA, USA). For double-labeling studies, tissue was then incubated overnight at 4 °C with antibodies raised in mouse to Olig2 (1:200; R and D Systems Cat# BAF2418; RRID:AB_2251803; R&D Systems, Minneapolis, MN, USA), calcitonin gene-related peptide (CGRP) (1:250; Sigma–Aldrich Cat# C7113; RRID:AB_259000; Sigma–Aldrich, St. Louis, MO, USA), CD45 (1:2000; AbD Serotec Cat# MCA43GA; RRID:AB_566759; Bio-Rad, Oxford, UK), S-100 (1:100; Millipore Cat# MAB079-1; RRID:AB_571112; Merck Millipore, Watford, UK) or PGP 9.5 (1:1000; UltraClone Cat# RA95101; RRID:AB_2313685; UltraClone, Histon, Cambridge, UK) or with isolectin B4 (IB4) (1:4000; Molecular Probes Cat# I21411; RRID:AB_2314662; Thermo Fischer Scientific, Waltham, MA, USA); diluted in PBST and 5% NDS. The sections were next incubated with a donkey anti-mouse secondary antibody, conjugated to fluorescein isothiocyanate (FITC) (1:300; Jackson ImmunoResearch Labs Cat# 715-095-150; RRID:AB_2340792i; Jackson ImmunoResearch Laboratories Inc., West Grove, PA, USA) diluted in PBST containing 1.5% NDS for 90 min at room temperature prior to being mounted and coverslipped as described above. Immunohistochemical controls for XCR1 were performed by liquid-phase preabsorption of the primary antibody with its respective ligand (10 nmol/ml). Images were acquired with a Zeiss Axioplan 2 imaging fluorescence microscope, fitted with a HBO 50 mercury lamp. Image acquisition and processing were performed with Image Pro-Plus (v5.1, Media Cybernetics, Rockville, MD, USA). For quantitative analysis of the constricted nerves, the specimen was divided into three regions (proximal, middle and distal; Fig. 1B) and the percentage area of positive immunofluorescence within the nerve was calculated for each region. The percentage area of positive immunofluorescence within the proximal and distal sections of the nerve was calculated over a distance of 500 µm from the most proximal or distal suture. Quantification was undertaken of a single section in each animal; analysis was carried out blind to the recovery period (i.e., 3 or 11 days) following CCI. Confocal images were obtained using a Nikon A1 confocal microscope.

#### XCR1 and glutamatergic synaptic terminals

In an additional group of animals XCR1 in the spinal trigeminal subnucleus caudalis (Vc) was localized with the markers of glutamatergic synaptic terminals vesicular glutamate transporter 1 and 2 (VGlut1 and VGlut2, respectively). Naïve adult male Wistar rats (*n* = 3; 200–250 g) were deeply anesthetized and perfused transcardially with PFA. The brainstems were dissected out, post-fixed in the same PFA solution for 2 h at 4 °C and transferred into PBS containing 0.01% azide for a minimum of 24 h. Serial 50-μm transverse brainstem sections were incubated for 72 h at 4 °C in PBS containing rabbit anti-XCR1 (1:250; Abcam Cat# ab67342; RRID:AB_2217066; Abcam, Cambridge, UK) and guinea-pig anti-VGlut1 (1:2500; Millipore Cat# AB5905; RRID:AB_2301751; Merck Millipore, Germany) or guinea-pig anti-VGlut2 (1:5000; Millipore Cat# AB2251; RRID:AB_1587626; Merck Millipore, Germany) antibodies. Appropriate direct secondary antibodies were applied, sections were mounted with Gel Mount aqueous mounting medium (Sigma–Aldrich, UK) and visualized using a fluorescence microscope. Images were captured using an inverted confocal microscope (LSM 700; Carl Zeiss Microscopy, USA) in conjunction with Zen 2.1 (Black) software (Carl Zeiss Microscopy, USA).

#### Reverse transcriptase–polymerase chain reaction (RT–PCR)

To determine the expression of rat XCR1 mRNA, adult male Sprague–Dawley rats (*n* = 2; 225–250 g) underwent mental nerve CCI as described above, and after 3 days were culled by pentobarbital (500 mg/kg, i.p.; J M Loveridge Ltd, UK). The right and left mental nerves and brainstem were removed and stored at −80 °C. Total RNA was isolated from rat mental nerve and brainstem using TRI REAGENT (Sigma–Aldrich, USA) following the manufacturer’s instructions. GAPDH was used as the housekeeping gene. The cDNA was amplified using the following primer sequences:

XCR1: sense AGCTGGGGTCCCTACAACTT, anti-sense GACCCCCACGAAGACATAGA;

GAPDH: sense AAATGGTGAAGGTCGGTGTGAAC, anti-sense CAACAATCTCCACTTTGCCAC.

The PCR cycles were performed at 94 °C for 1 min, annealing at 60 °C for 2 min and extension at 72 °C for 3 min for a total of 35 cycles. A 1% agarose gel containing ethidium bromide was used to separate the PCR fragments and the gel was visualized and photographed using an imaging system (Syngene, Cambridge, UK).

### Assessment of c-Fos, pERK and pp38 activation following application of XCL1

#### *In vitro* activation of c-Fos, pERK and pp38

In order to identify the effect of XCL1 and its antagonist viral CC chemokine macrophage inhibitory protein-II (vMIP-II; Shan et al., 2000) on the activity of c-Fos, pERK and pp38, naïve male Wistar rats (*n* = 16; 70–80 g) were irreversibly anesthetized with pentobarbital (40–50 mg/kg, i.p.; Sigma–Aldrich, UK) and then received 0.1 ml Lignol (2.0% lignocaine w/v with adrenalin; Dechra Veterinary Products Ltd, UK) s.c. into the neck scruff at the base of the skull to reduce incisional sensory activation. The rats were then perfused transcardially with ice-cold heparinized (0.1%) artificial cerebrospinal fluid (aCSF; in mM: NaCl, 128; KCl, 1.9; KH_2_PO_4_, 1.2; MgSO_4_, 1.3; CaCl_2_, 2.4; NaHCO_3_, 26; glucose, 10; pH 7.4) and the brainstem was removed, dissected free of meninges and placed into a plastic holding chamber containing aCSF maintained at a constant 37 °C using a water bath. The tissue was left to rest for 2 h in aCSF containing the sodium channel blocker tetrodotoxin (TTX) (1 μM; Tocris-Cookson, Bristol, UK) added to prevent non-specific neuronal activation and indirect conducted excitation. Brainstems were maintained for an additional 2 h in aCSF containing TTX plus XCL1 (0.1 µM; *n* = 4), vMIP-II (0.1 µM; *n* = 4), or XCL1 (0.1 µM) plus vMIP-II (0.1 µM; *n* = 4). Controls (*n* = 4) were incubated in aCSF and TTX only. The tissue was then retained in aCSF containing TTX for 1 h, before fixation overnight in 4% PFA prior to processing for immunohistochemistry.

#### Immunohistochemistry

Tissue was cut into serial 50-μm transverse sections, between obex and ∼1600 µm caudal to obex, using a vibratome (Leica Microsystems, Wetzlar, Germany). Sections were blocked in 0.1% PBST containing 10% NDS for 1 h at room temperature and then incubated in one of: rabbit anti-pp38 (1:300; Cell Signaling Technology Cat# 9211; RRID:AB_331641; Cell Signaling Technology, Danvers, MA, USA); rabbit anti-pERK (1:500; Cell Signaling Technology Cat# 4370; RRID:AB_2315112; Cell Signaling Technology, Danvers, MA, USA); or goat anti c-Fos (1:1000; Santa Cruz Biotechnology Cat# sc-52-G; RRID:AB_2629503; Santa Cruz Biotechnology Inc., Dallas, TX, USA) diluted in 0.1% PBST and 5% NDS overnight at 4 °C or room temperature. Sections were next incubated with appropriate secondary antibodies (1:1000) for 2 h, mounted with Vectashield mounting medium (Vector Laboratories, CA, USA) and cover-slipped. For dual-labeling studies, brainstem sections were incubated overnight with antibodies to the neuronal marker NeuN (rabbit anti-NeuN, 1:1000; Millipore Cat# MAB377; RRID:AB_2298772; Millipore, Billerica, MA, USA), the microglial marker ionized calcium-binding adapter molecule 1 (Iba1) (goat anti-Iba1, 1:500; Abcam Cat# ab5076; RRID:AB_2224402; Abcam, Cambridge, UK), or the astrocyte marker glial fibrillary acidic protein (GFAP) (mouse anti-GFAP, 1:1000; Millipore Cat# IF03L; RRID:AB_212974; Merck Millipore, Watford, UK), and then with appropriate secondary antibody. Controls included omission of the primary or secondary antibodies. Immunolabeling was visualized using an inverted confocal microscope (LSM 700; Carl Zeiss Microscopy, USA) in conjunction with Zen 2.1 (Black) software (Carl Zeiss Microscopy, USA). For semi-quantification of pp38 expression levels, the mean intensity of staining from a fixed-size region of Vc visualized at low magnification (×10) was calculated using ImageJ software and plotted as a total mean ± standard error of the mean (SEM) value calculated across a portion of Vc running from obex to 1600 μm caudal to obex (eight sections per animal at 200-μm intervals throughout Vc). For quantification of c-Fos and pERK, positive nuclei within the trigeminal subnucleus caudalis were counted from obex to 1600 μm caudal to obex (eight sections per animal at 200-μm intervals throughout Vc). Sections were counted blind by the same investigator.

#### Electrophysiology

Female Wistar rats (*n* = 6; 65–75 g) were deeply anesthetized and perfused transcardially with ice-cold aCSF containing sucrose. The brainstem was removed quickly and placed in oxygenated (95% O_2_/5% CO_2_) ice-cold standard aCSF to remove meninges. The brainstem was trimmed rostrocaudally to isolate an area 2–3 mm caudal to obex (including Vc) and embedded in 3% agar solution (Alfa Aesar, Haverhill, MA, USA). Transverse brainstem slices (350 µm) were cut using a vibratome (Leica VT1000S, Leica Microsystems, Germany) and placed into ice-cold, oxygenated aCSF. Slices were transferred to a holding chamber, where they were submerged in oxygenated aCSF maintained at 35 °C and incubated for 1 h. The slices were then incubated for 2 h with one of the following, diluted in aCSF: mouse recombinant XCL1 (0.1 µM; Sigma–Aldrich, UK); XCL1 antagonist vMIP-II (0.1 µM; R&D Systems, Minneapolis, MN, USA); normal aCSF; or vMIP-II (0.1 µM) plus XCL1 (0.1 µM) prior to the electrophysiological recordings.

For recordings, brainstem slices were transferred to a custom-built Perspex chamber that maintained tissue in an interface between a warm (32 °C), humidified carbogen (95% O_2_/5% CO_2_) environment and aCSF at an average flow rate of 1–1.5 ml/min. Extracellular field recordings of baseline and drug-induced subthreshold rhythmic activity were made using borosilicate glass microelectrodes (Harvard Apparatus, Kent, UK; 10–20 MΩ) filled with normal aCSF and placed at a depth of 15 µm into the superficial laminae of trigeminal Vc. Voltage waveforms were recorded and amplified (×10) by an Axoclamp 2A system (Molecular Devices, CA, USA), with further amplification (×1000) provided by a Neurolog NL106 module (Digitimer, Welwyn Garden City, UK). The voltage signals in all experiments were filtered using a low-pass band filter setting of 40 Hz (Neurolog NL125; Digitimer). Voltage waveforms were digitized at 5 kHz and captured for further analysis with Spike 2 software (Cambridge Electronic Design, Cambridge, UK). To extract characteristics of subthreshold low-amplitude voltage oscillations, power spectra were generated using 1-second epochs and the amplitude of the peak frequency measured to give the power of the oscillation. Power amplitude (measured as maximum peak height in spectra) and area values (calculated as the area under the curve for two cursors set at 4 and 12 Hz in spectra) were derived from an average of five consecutive 1-second epochs. The sampling rate was 5 kHz, which was divided by the 8192 points in the fast Fourier transform, to provide an overall resolution of 0.6 Hz.

### Statistical analysis

Data analysis and statistical comparisons were performed using GraphPad Prism (version 5.0d; RRID:SCR_002798; GraphPad Software, Inc., La Jolla, CA, USA). Analysis of variance (ANOVA) with a post-hoc Tukey’s multiple comparisons test was performed to assess the difference in the level of XCR1 expression at the injury site between experimental groups. A one-way ANOVA with Dunnett’s post-hoc test was used to analyze parameters of power amplitude and power area and levels of c-Fos, pERK and pp38. Data values are expressed as the mean ± SEM. Values of *p* < 0.05 were considered statistically significant.

## Results

### XCR1 is expressed in the peripheral nervous system

Immunohistochemical labeling for XCR1 was present in the rat mental nerve 3 days following CCI (Fig. 1); no labeling was present in the mental nerves of sham-operated or naïve animals. Two types of XCR1 labeling were observed in the injured mental nerve: beaded fiber-like staining indicative of labeling within a nerve fiber, and irregular dense punctate labeling (Fig. 1A). At 3 days post injury, immunoreactive labeling for XCR1 was present in the proximal, middle and distal regions of the constricted nerve (Fig. 1B): quantitative image analysis revealed significantly higher levels of XCR1 immunoreactivity in the middle region of the constricted mental nerve (6050 ± 2292 µm^2^ [SEM]), compared with the proximal (2033 ± 453 µm^2^) and distal (282 ± 153 µm^2^) regions (*p* < 0.05; Fig. 1D). Levels of XCR1 labeling appeared higher 3 days after CCI, compared with 11 days after (Fig. 1C), with quantitative analysis revealing a statistically significant difference between the two recovery periods (2788 ± 567 µm^2^ [3 days] vs 218 ± 135 µm^2^ [11 days]: *p* < 0.0001; Fig. 1D). The quantity of XCR1 labeling after 3 days was significantly higher in the CCI group than in the Sham group (no labeling) (*p* < 0.0001). Positive XCR1 labeling was abolished following liquid-phase pre-absorption of the antibody with its respective antigen, indicating specificity of the XCR1 antibody (Fig. 1A). The time course of increased XCR1 expression seen here correlates with the development of spontaneous activity at sites of trigeminal nerve injury (Bongenhielm and Robinson, 1996, Bongenhielm and Robinson, 1998, Yates et al., 2000).

**Fig. 2:**
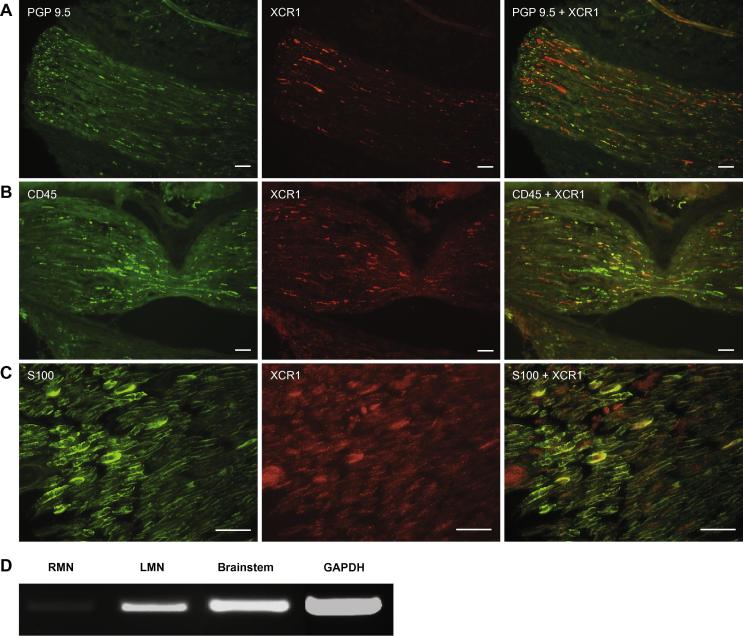
XCR1 co-exists with the neuronal marker PGP9.5, the leukocyte common antigen CD45 and the Schwann cell marker S-100 in injured mental nerve, 3 days following chronic constriction injury (CCI). (A) Immunofluorescent labeling of PGP9.5, highlighting viable nerve axons (green), XCR1 within the mental nerve (red), and co-localization of XCR1 within PGP9.5-labeled axons of the mental nerve (yellow). Scale bars = 100 μm. (B) Expression of CD45-positive cells expressing XCR1-like immunoreactivity (yellow) in injured mental nerve. Scale bars = 100 μm. (C) Co-localization of XCR1 with structures positively labeled with S-100 (yellow). Scale bars = 50 μm. (D) 3 days following CCI, RT–PCR revealed much higher levels of XCR1 mRNA in injured left mental nerve (LMN) compared with the uninjured right mental nerve (RMN), where mRNA was barely detectable; XCR1 mRNA was also present in the brainstem.

As illustrated in Fig. 2A, dual labeling of the mental nerve for XCR1 and the neuronal marker PGP9.5 revealed that while a proportion of XCR1 labeling was co-localized within PGP9.5-labeled nerve fibers, there was a substantial proportion that was not. Further labeling of the mental nerve with the leukocyte common antigen CD45 (Fig. 2B) and Schwann cell marker S-100 (Fig. 2C) indicated that XCR1 also showed some degree of co-localization with these two cell types.

In addition, quantitative RT–PCR analysis of tissue collected from rats 3 days following CCI revealed that the level of XCR1 mRNA in the injured (left) mental nerve was much higher when compared with the uninjured (right) side, where mRNA was barely detectable (Fig. 2D). XCR1 mRNA was also detected in the brainstem of CCI rats (Fig. 2D).

### XCR1 is expressed in the central nervous system

**Fig. 3:**
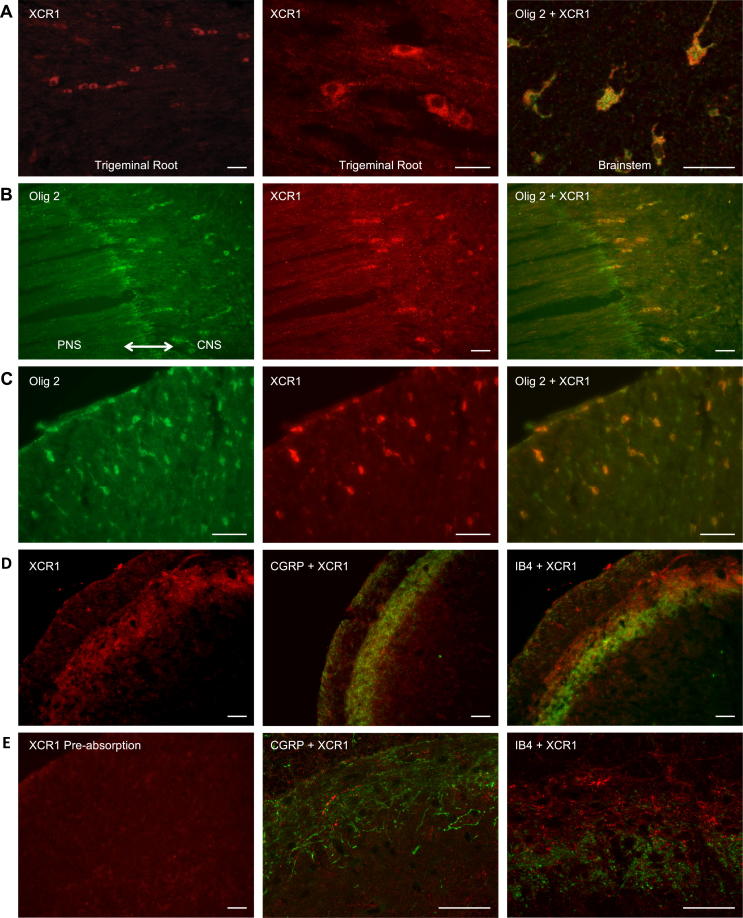
XCR1 is expressed in the trigeminal root and brainstem. (A) Immunofluorescent XCR1 positively labeled cells (red) in the trigeminal root (red) and co-localized in oligodendrocytes (yellow) in the brainstem. (B) Positive co-localization of XCR1 with oligodendrocyte marker Olig2, in the central part of the transition zone. (C) XCR1 immunoreactive-positive cells (red) in the white matter of the brainstem, co-localized (yellow) with Olig2-positive cells (green). (D) XCR1 is expressed in the Vc region of the trigeminal nucleus, showing expression within laminae I and IIo and in the same region as CGRP, but not in laminae IIi or in the same region as IB4. (E) Following pre-absorption of the XCR1 primary antibody with its respective antigen, no XCR1 labeling was observed. Confocal analysis showed XCR1 does not co-localize with CGRP- or IB4-labeled fibers. All scale bars = 50 μm.

A population of XCR1-positive cells was observed in a region of the trigeminal root, central to the transition zone (the boundary between the peripheral and central nervous systems) (Fig. 3A, B). This population of XCR1-positive cells also expressed the oligodendrocyte marker Olig2 thus indicating that these cells were oligodendrocytes (Fig. 3B). In addition, immunohistochemical staining of central nervous tissue revealed XCR1-positive labeling in two distinct areas of the brainstem, the white matter and the trigeminal nucleus. In the white matter, XCR1 was specifically co-localized with Olig2, indicating expression in oligodendrocytes (Fig. 3A, C); there was no evidence of XCR1 expression in either microglia or astrocyte glial cells. In the trigeminal nucleus, XCR1 labeling was confined to subnucleus caudalis (Vc), with no expression observed in either interpolaris or oralis. XCR1 appeared to be localized to nerve terminals in Vc, in the lamina I and IIo region that contains CGRP-positive primary afferent terminals (Fig. 3D); however, when observed using high-magnification confocal microscopy there was little evidence of colocalization of XCR1 and CGRP (Fig. 3E). The region of XCR1 labeling was distinct from that for isolectin IB4, seen in the lamina IIi region of Vc, as illustrated in Fig. 3D, E. Positive XCR1 labeling was abolished following liquid-phase pre-absorption of the antibody with its respective antigen, indicating specificity of the XCR1 antibody (Fig. 3E).

### XCR1 is expressed in the superficial laminae of trigeminal subnucleus caudalis and co-exists with VGlut2

**Fig. 4:**
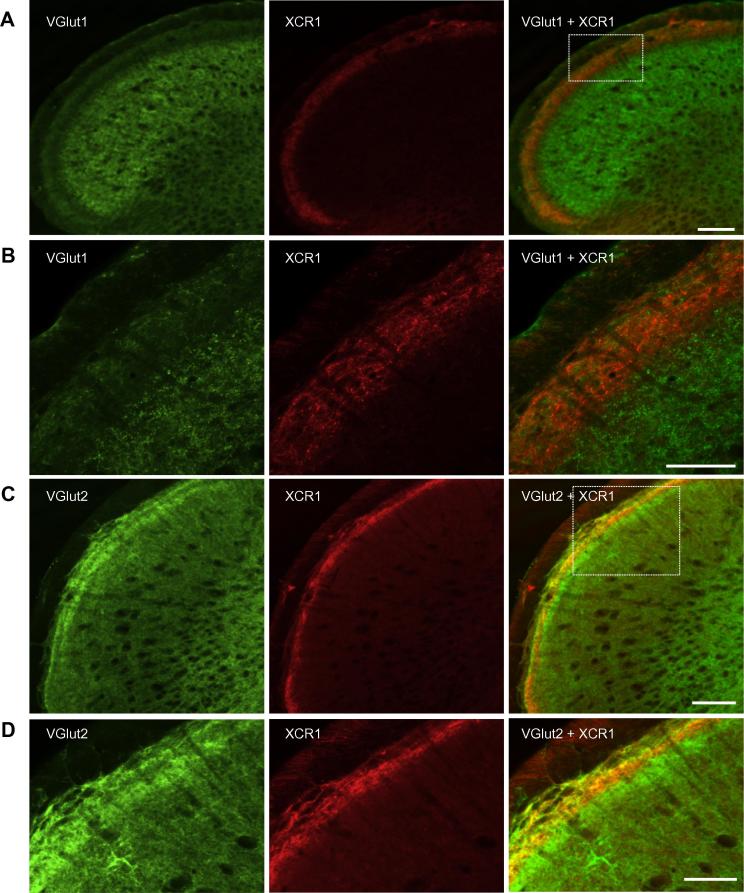
XCR1 immunoreactivity in Vc was found to overlap extensively with the vesicular glutamate transporter 2 (VGlut2). (A–D) Confocal images of 50-μm transverse sections of brainstem cut caudally from obex. (A, B) Co-localization of XCR1 (red) and VGlut1 (green) in the caudal brainstem. (C, D) Co-localization of XCR1 (red) and VGlut2 (green) in the caudal brainstem. (B, D) Show enlargement of squared boxes in A and C, respectively. In (A–D) the single staining for each antibody and the merged image are shown from left to right and double staining appears in yellow. All scale bars = 100 μm.

Confocal imaging of Vc in the caudal brainstem sections of naïve rats revealed intense labeling for XCR1 in the most superficial layers of Vc (Fig. 4). The diffuse pattern of labeling was indicative of an axonal or fiber localization rather than in cell bodies. In double-labeling studies with the vesicular glutamate transporters VGlut1 or VGlut2 (which mark glutamatergic synaptic terminals), labeling for XCR1 in Vc showed colocalization with VGlut2 in superficial layers whereas VGlut1 staining was distributed to deeper regions with little evidence of overlap (Fig. 4A–D).

### XCL1-mediated induction of c-Fos, pERK and pp38 within trigeminal subnucleus caudalis

**Fig. 5:**
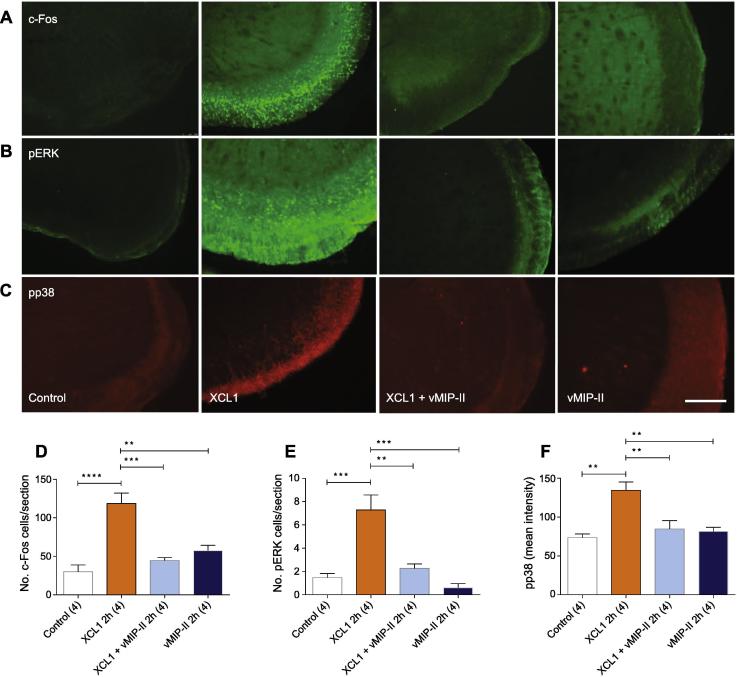
XCL1 increases expression of c-Fos, pERK and pp38 in Vc that is blocked by the XCR1 antagonist viral CC chemokine macrophage inhibitory protein-II (vMIP-II). Distribution of immunolabeling for (A) c-Fos, (B) pERK and (C) pp38 in Vc following a 2-hour incubation with drug-free aCSF (control), XCL1, XCL1 + vMIP-II, or vMIP-II. c-Fos, pERK and pp38 labeling is localized to the most superficial layers of Vc and is more pronounced in XCL1-exposed Vc tissue. Incubation of trigeminal brainstem slices with XCL1 (2 h) resulted in an increased activation of c-Fos (D), pERK (E) and pp38 (F) in the superficial layers of Vc. vMIP-II blocked XCL1-induced activation of c-Fos (D), pERK (E) and pp38 (F) in the superficial layers of Vc. Numbers in parenthesis indicate animals used. ^∗∗^*p* < 0.01, ^∗∗∗^*p* < 0.001, ^∗∗∗∗^*p* < 0.0001 (ANOVA with Dunnett’s post-hoc test). Data are expressed as the mean ± SEM. Scale bar = 500 μm.

**Fig. 6:**
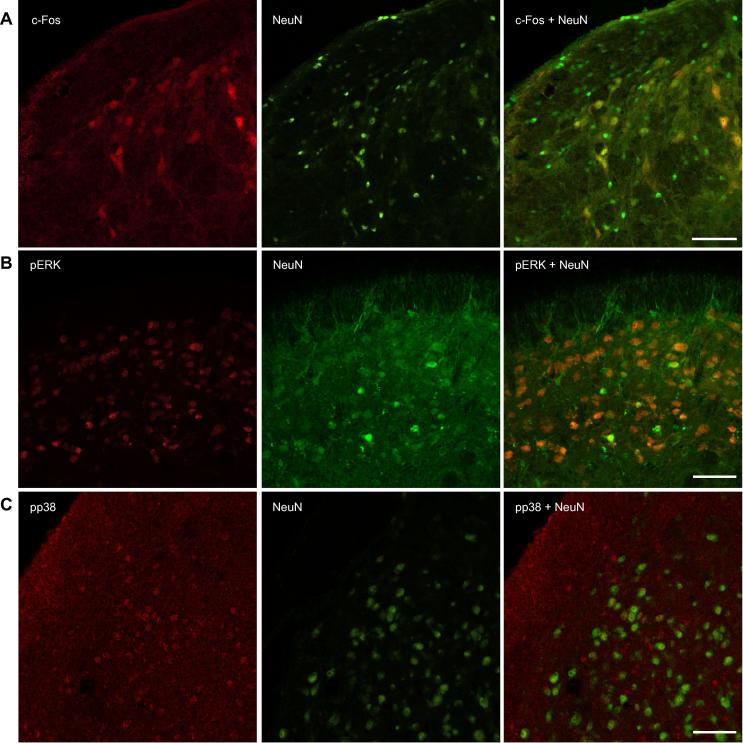
c-Fos, pERK and pp38 are expressed in neuronal cells in Vc. Immunolabeling for c-Fos (A), pERK (B) and pp38 (C) co-localizes with neuronal marker NeuN in Vc. Representative figures are from brainstem slices incubated for 2 h in aCSF containing TTX plus XCL1 (see Methods) prior to processing for immunohistochemistry. (A–C) Confocal images of 50-μm transverse sections of brainstem caudal to obex. (A) Co-localization of c-Fos (red) and NeuN (green) in Vc. (B) Co-localization of pERK (red) and NeuN (green) in Vc. (C) Co-localization of pp38 (red) and NeuN (green) in Vc. In (A–C) the single staining for each antibody and the merged image are shown from left to right; co-localization appears in yellow. Scale bars = 10 μm.

Expression of c-Fos, pERK and pp38 was much greater following direct exposure of brainstem tissue to XCL1, compared with that in control tissue. As illustrated in Fig. 5A–C, immunofluorescence for c-Fos, pERK and pp38 following incubation of the brainstem *in vitro* for 2 h in aCSF containing XCL1, revealed localized and profuse labeling in the superficial laminae (laminae I–II) of Vc. XCL1-induced immunolabeling for c-Fos (118.6 ± 11.1 cells/section), pERK (7.29 ± 1.05 cells/section) and pp38 (mean intensity 134.4 ± 8.9) was significantly increased when compared with that in matched Vc tissue incubated with XCL1 in presence of the XCL1 antagonist vMIP-II (44.5 ± 3.0 cells/section [*p* < 0.001], 2.26 ± 0.31 cells/section [*p* < 0.01] and mean intensity 84.7 ± 8.7 [*p* < 0.01], respectively), vMIP-II alone (57.4 ± 5.6 cells/section [*p* < 0.01], 0.60 ± 0.29 cells/section [*p* < 0.001] and mean intensity 81.6 ± 4.3 [*p* < 0.01], respectively) or untreated controls incubated in drug-free aCSF (29.9 ± 7.30 cells/section [*p* < 0.0001], 1.47 ± 0.28 cells/section [*p* < 0.001] and mean intensity 73.6 ± 3.90 [*p* < 0.01], respectively) (Fig. 5D–F). Since *in vitro* Vc tissue was co-incubated with TTX, which effectively uncoupled axonally conducted excitation, it is likely that expression of c-Fos, pERK and pp38 was directly induced by XCL1 rather than by indirect non-specific excitation. Expression of c-Fos, pERK and pp38 was most pronounced in the superficial regions of Vc, though it clearly extended to deeper Vc laminae (laminae III–IV; Fig. 5). Using a double-labeling protocol that combined c-Fos, pERK and pp38 immunofluorescence with that for the neuronal marker NeuN, microglial marker Iba1 and astrocyte marker GFAP, positive labeling for c-Fos, pERK and pp38 was only colocalized with that for NeuN (Fig. 6).

### XCL1 acting via XCR1 increases neuronal excitability in trigeminal subnucleus caudalis

**Fig. 7:**
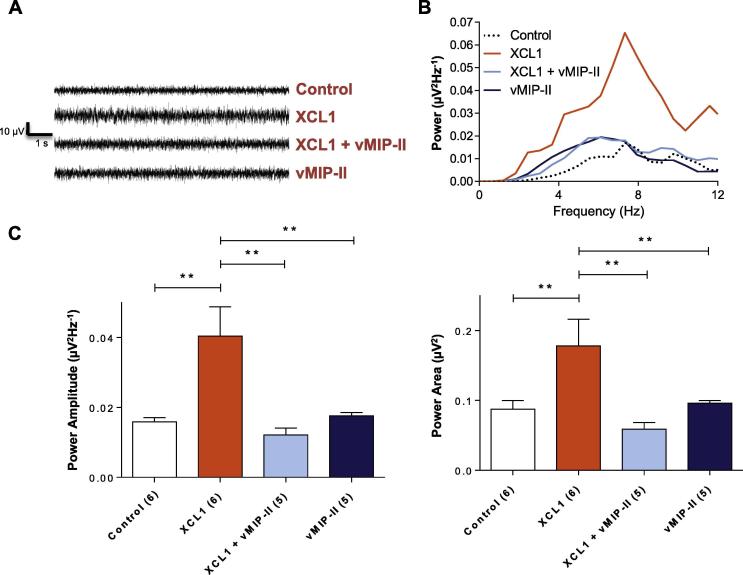
XCL1 acting via XCR1 increases neuronal excitability in Vc. (A) Exemplar raw data trace from a single trigeminal brainstem slice showing: low-level spontaneous subthreshold voltage oscillations recorded in Vc *in vitro* in control aCSF (top trace); enhanced ongoing 4- to 12-Hz oscillatory activity in a single brainstem slice incubated in XCL1 (0.1 μM, 2 h) (upper middle trace); reduced intensity of XCL1-induced oscillatory activity by co-incubation of XCL1 and the antagonist vMIP-II (0.1 μM, 2 h) (lower middle trace). (B) Power spectra derived from raw data shown in (A) of low-amplitude rhythmic oscillations reveal a dominant frequency within 4- to 12-Hz frequency band and enhanced 4- to 12-Hz activity after exposure to XCL1 that is reduced by vMIP-II. Note that, relative to the drug-free control, MIP-II alone did not enhance baseline oscillatory activity. (C) The peak power amplitude and power area of the 4- to 12-Hz rhythmic activity was significantly increased within Vc in slices bathed in XCL1 (*p* < 0.01); this effect was blocked by vMIP-II. Data are expressed as mean ± SEM and the number of slices used for each is shown in parenthesis on the *x*-axis (ANOVA with Dunnett’s post-hoc test).

Functional electrophysiology recordings showed that in rat brainstem slices bathed in control aCSF for 2 h, a minimal level of subthreshold spontaneous rhythmic (4–12 Hz) excitatory activity was recorded within Vc (Fig. 7A, B). In brainstem slices pre-incubated and bathed in XCL1 for 2 h, an increased level of ongoing 4–12 Hz neuronal activity was recorded within Vc; this activity was blocked by the potent XCL1 antagonist vMIP-II (Fig. 7A, B). Data quantification revealed that the parameters of peak power amplitude and integrated power area of the 4–12 Hz rhythmic activity were significantly increased in Vc of brainstem slices bathed in XCL1 when compared with control (Fig. 7C; *p* < 0.01). Compared with control values (expressed as 10^−6^ V^2^) of 0.016 ± 0.001 for power amplitude and 0.087 ± 0.012 for power area (*n* = 6), the corresponding values increased to 0.040 ± 0.008 and 0.178 ± 0.038, respectively (both *p* < 0.01), in the presence of XCL1 (2 h, *n* = 6). This low-amplitude subthreshold oscillatory behavior possessed characteristics similar to those induced by the epileptogenic drug 4-AP (25 µM), as previously reported for spinal dorsal horn (Chapman et al., 2009). The XCL1-induced increases in power amplitude and power area were blocked by vMIP-II (0.012 ± 0.002 10^−6^ V^2^ and 0.059 ± 0.009 10^−6^ V^2^, respectively [*n* = 5]; both *p* < 0.01) (Fig. 7C). Neither vMIP-II nor XCL1 had an effect on the dominant frequency of spontaneous rhythmic activity, which remained constrained to the 4–12 Hz range (Fig. 7B).

## Discussion

This is the first study to identify XCR1 in the trigeminal system, to demonstrate that XCR1 is upregulated at sites of nerve injury, and to describe a role for the XCL1-XCR1 axis in modulating intracellular signaling and neuronal excitability.

### XCR1 is upregulated at sites of nerve injury

We have demonstrated that the expression of XCR1 in the rat mental nerve is elevated 3 days following CCI, compared with 11 days post-CCI and sham controls. At this site XCR1 co-exists with neuronal marker PGP9.5, leukocyte common antigen CD45, and Schwann cell marker S-100. The time course of altered XCR1 expression relates closely to that of the development of spontaneous (ectopic) activity at sites of trigeminal nerve injury (Bongenhielm and Robinson, 1996, Bongenhielm and Robinson, 1998, Yates et al., 2000). Ectopic activity at these sites plays a role in the development of pain following nerve injury (Devor and Seltzer, 1999). A number of modulators of neuronal excitability show increased expression at trigeminal nerve injury sites, including neuropeptides, ion channels, nitric oxide synthase and TRP channels (Bird et al., 2002, Bird et al., 2003, Davies et al., 2004, Davies et al., 2006, Biggs et al., 2007), and other chemokines and their receptors (discussed below). Many of these molecules have been implicated in the development of spontaneous activity, but the precise mechanisms are not yet established. Upregulation of XCR1 in a number of cell types at the injury site could also implicate the XCL1-XCR1 axis in the development of ectopic activity, via neural–immune and/or neural–glial interactions; thus, this axis has potential to contribute to the development of neuropathic pain.

At the nerve injury site, XCR1 was present in nerve fibers, CD45-positive leucocytes and Schwann cells. Other chemokine receptors have also been reported in a range of cell types in the peripheral nervous system, and shown to be upregulated following nerve injury. For example, the CXCL12 receptor CXCR4 has been found within sciatic nerve fibers where it is partially colocalized with CGRP-positive axons (Reaux-Le Goazigo et al., 2012). Increased expression of CXCR4 has been observed in macrophages following sciatic nerve injury (Dubový et al., 2007, Dubový et al., 2010), and CXCR4 activation contributes to persistent pain via regulating the excitability of peripheral nociceptive neurons (Yang et al., 2015). The CCL2 receptor CCR2 is also expressed in macrophages – at the injury site and in the DRG following nerve injury (Abbadie et al., 2003) – and has been implicated in the development of neuropathic pain. Thus both the CXCL12-CXCR4 and the CCL2-CCR2 chemokine axes have been shown to contribute to peripheral mechanisms of nociception and neuropathic pain. Similarly, altered expression of XCR1 in neurons and immune cells at sites of nerve injury indicates the potential of the XCL1-XCR1 axis to influence development of neuropathic pain via a peripheral mechanism.

### XCR1 is expressed in glial cells involved in myelination

The expression of XCR1 in Schwann cells at the nerve injury site, and in oligodendrocytes in the trigeminal root and white matter of the brainstem is of potential interest. These cells have a well-established role in myelination but there also is growing evidence that they have an important role in regulating local immune responses and can contribute to the development of inflammatory peripheral neuropathies (Ydens et al., 2013) and a range of central nervous system (CNS) disorders. Chemokine expression has been reported in Schwann cells (eg MCP-1/CCL2) and linked to neuroinflammatory disease (eg Guillain–Barré syndrome) (Orlikowski et al., 2003). Several chemokine receptors (eg CXCR1, CXCR2, CXCR3) are expressed in oligodendrocytes and show increased expression in MS, stroke and amyotrophic lateral sclerosis (Omari et al., 2005), and have been implicated in disorders in which myelin damage is associated with immune activation in the CNS. The particular association between XCR1 and myelinating glia suggests a potential role for XCR1 in diseases linked with myelination disorders.

### XCR1 is expressed in nerve terminals in the outer laminae of Vc

In Vc, XCR1 immuno-positive labeling was distributed most strongly across the outer superficial laminae and was found only sparsely in deeper laminae. The lack of clearly labeled cell bodies and the diffuse pattern of XCR1 immuno-positive staining were suggestive of an association with axonal arborizations, fibers or local terminals. In relation to nociceptive primary afferent terminals, no association or overlap was found between immunolabeling for IB4-positive terminals and XCR1, thus excluding a strong association for XCR1 with this class of non-peptidergic C-afferent. Immuno-positive staining for both XCR1 and CGRP co-existed within lamina I and IIo but there was little evidence of clear co-localization so it is not possible to associate XCR1 with CGRP-expressing peptidergic C-afferents. Examination of the expression patterns for either vGlut1 or vGlut2 transporters indicated a clear regional separation with the highest density of immunolabeling for vGlut1 across deep laminae whereas vGlut2 was within superficial laminae, particularly laminae II. Double-immunolabeling with XCR1 indicated a strong co-localization with vGlut2 but not vGlut1. Thus these data show that XCR1 is expressed in vGlut2- but not vGlut1-, CGRP-, or IB4-containing terminals. This is consistent with previous studies reporting that few vGlut2-containing terminals express either CGRP or IB4, and that these three markers are typically expressed in separate populations of terminals in dorsal horn and Vc (Todd et al., 2003, Morris et al., 2005, Hegarty et al., 2010). In spinal dorsal horn, many vGlut2-containing terminals originate from local glutamatergic interneurons (Todd et al., 2003). Taken together, this suggests that XCR1 may be expressed in terminals of A-delta afferents, C-fiber afferents that are non-peptidergic and non-IB4 binding, and/or within excitatory interneurons. While the presence of chemokine receptors in the spinal cord and Vc is well documented, their expression is generally reported in microglia and/or neuronal cell bodies – eg CCR2 (Abbadie, 2005, Zhang et al., 2012) and CX3CR1 (Lindia et al., 2005, Kiyomoto et al., 2013). Thus the pattern of labeling for XCR1 appears somewhat different to that reported for other chemokine receptors. The only previous study to report XCR1 expression in the CNS (Zychowska et al., 2016) describes increased levels in a mouse model of type 1 diabetes (streptozotocin model). The study reported XCR1 to be present in neuronal cell bodies in the lumbar spinal cord but did not provide further detail as to the location of the XCR1-containing cell bodies.

### XCL1 induces physiological and cellular indices of central sensitization in Vc

The chemokine XCL1 is produced in infectious and inflammatory processes but its role in mechanisms of central sensitization in Vc is unknown. Two different *in vitro* methodologies indicated that XCL1 could potentially and substantially drive central sensitization within trigeminal dorsal horn circuitry. Firstly, c-Fos, pERK and pp38 immunolabeling localized to laminae I–II of Vc was significantly increased in trigeminal brainstem tissue exposed to XCL1. The colocalization of these markers with neurons in Vc provides further evidence that XCR1 is expressed on terminals of Vc interneurons (discussed above). Expression of c-Fos, pERK and pp38 in Vc is used widely as a marker of central sensitization in models of persistent orofacial pain (Anton et al., 1991, Hathaway et al., 1995, Worsley et al., 2014) and, in spinal cord, expression of these markers is induced by noxious stimulation or tissue injury (Hunt et al., 1987, Gao and Ji, 2009, Ji et al., 2009). Secondly, XCL1 directly and significantly enhanced spontaneous rhythmic 4–12 Hz low-amplitude voltage oscillations recorded extracellularly in the Vc region. Spontaneous subthreshold neuronal activity of this type has been characterized in the spinal dorsal horn (Chapman et al., 2009, Kay et al., 2016). This behavior can be induced by 4-aminopyridine (4-AP), a convulsant used in a model of spinal hyperexcitability (Ruscheweyh and Sandkühler, 2003). These electrophysiological functional data reveal that application of XCL1 induces increased spontaneous low-threshold activity, which may represent a form of hyperexcitability within the Vc nociceptive circuitry. Spontaneously hyperactive neuronal discharges are an established characteristic of chronic pain and a known driver for central sensitization (Suzuki and Dickenson, 2006). Enhanced expression of molecular markers of pain and increased neuronal excitability were both significantly attenuated by the specific XCR1 antagonist vMIP-II, thereby directly linking these effects of XCL1 to its cognate receptor. There is evidence that application of CXCL12, CCL2 or CX3CL1 to the spinal cord (intrathecally) and/or Vc (intracisternally) produces behavioral changes such as mechanical allodynia and thermal hyperalgesia (Abbadie, 2005, Kiyomoto et al., 2013), indicating a role for these chemokines in modulation of central nociceptive processing. The data we present here infer a putative role for the XCL1-XCR1 axis in central modulation of nociceptive processing and the development of central sensitization in the trigeminal pain system. However, further studies of the effects of XCL1 on Vc synaptic excitation and sensory afferent inputs will be required to confirm this.

The data in this paper were obtained using different strains of rats, which reflect the working practices of the two labs, our established protocols and the specific requirements for the techniques used. For example, the slice preparation experiments are most reliable in young animals (65–80 g), whereas the CCI injury requires the use of larger animals (225–250 g). However, of particular relevance in relation to this is that the laminar distribution of XCR1 in Vc and that of XCL1-mediated induction of c-Fos, pERK and pp38 across these variations are the same, thus demonstrating further the robust nature of the data, with reproducibility across strains and ages.

In conclusion, we have demonstrated that XCR1 is upregulated at sites of trigeminal nerve injury with a time course that correlates with the development of spontaneous activity at these sites. In Vc, XCR1 immunoreactivity is present in the outer laminae in a manner consistent with XCR1 expression in terminals of A-delta afferents, C-fiber afferents that are non-peptidergic and non-IB4 binding, and/or excitatory interneurons. Exposure of trigeminal brainstem tissue to XCL1 induces expression of c-Fos, pERK and pp38 immunolabeling in laminae I–II of Vc, and induces increased spontaneous low-threshold activity indicative of hyperexcitability within the Vc nociceptive circuitry. Taken together the data from this study provide the first evidence that the XCL1-XCR1 axis may play a role within peripheral and central trigeminal pain pathways, and indicate that this axis may provide a potential target for novel analgesics.

## Author contributions

FMB with AEK and EVB conceived and supervised the project. EVB, CRC and VA carried out immunohistochemical studies for XCR1 and S-100, PGP9.5, CD45, CGRP, IB4 and Olig2 in mental nerve, trigeminal root and brainstem. IO and TI carried out immunohistochemical studies for XCR1 and vGlut1/2, c-Fos, pERK and pp38. TI carried out the electrophysiological studies. EVB, CRC, TI, IO, AEK and FMB contributed to the preparation of the manuscript.
